# Hydrocarbon-Degrading Bacteria Found Tightly Associated with the 50–70 μm Cell-Size Population of Eukaryotic Phytoplankton in Surface Waters of a Northeast Atlantic Region

**DOI:** 10.3390/microorganisms8121955

**Published:** 2020-12-09

**Authors:** Haydn Frank Thompson, Stephen Summers, Raif Yuecel, Tony Gutierrez

**Affiliations:** 1 Institute of Mechanical, Process and Energy Engineering (IMPEE), School of Engineering and Physical Sciences, Heriot-Watt University, Edinburgh EH14 4AS, UK; haydnft@gmail.com (H.F.T.); ssummers@ntu.edu.sg (S.S.); 2 The Singapore Centre for Environmental Life Sciences Engineering, Nanyang Technological University, Singapore 637551, Singapore; 3 Iain Fraser Cytometry Centre, Institute of Medical Sciences IMS, University of Aberdeen, Aberdeen AB25 2ZD, UK; R.Yuecel@exeter.ac.uk; 4 Exeter Centre for Cytomics (EXCC), College of Life and Environmental Sciences, University of Exeter, Exeter, EX4 4QD, UK

**Keywords:** hydrocarbonoclastic bacteria, *Marinobacter*, eukaryotic phytoplankton, micro-algae, phycosphere, fluorescence in situ hybridisation (FISH), flow cytometry, hydrocarbons, marine environment

## Abstract

The surface of marine eukaryotic phytoplankton can harbour communities of hydrocarbon-degrading bacteria; however, this algal–bacterial association has, hitherto, been only examined with non-axenic laboratory cultures of micro-algae. In this study, we isolated an operationally-defined community of phytoplankton, of cell size 50–70 μm, from a natural community in sea surface waters of a subarctic region in the northeast Atlantic. Using MiSeq 16S rRNA sequencing, we identified several recognized (*Alcanivorax*, *Marinobacter*, *Oleispira*, *Porticoccus*, *Thalassospira*) and putative hydrocarbon degraders (*Colwelliaceae*, *Vibrionaceae*) tightly associated with the phytoplankton population. We combined fluorescence in situ hybridisation with flow-cytometry (FISH-Flow) to examine the association of *Marinobacter* with this natural eukaryotic phytoplankton population. About 1.5% of the phytoplankton population contained tightly associated *Marinobacter*. The remaining *Marinobacter* population were loosely associated with either eukaryotic phytoplankton cells or non-chlorophyll particulate material. This work is the first to show the presence of obligate, generalist and putative hydrocarbonoclastic bacteria associated with natural populations of eukaryotic phytoplankton directly from sea surface water samples. It also highlights the suitability of FISH-Flow for future studies to examine the spatial and temporal structure and dynamics of these and other algal–bacterial associations in natural seawater samples.

## 1. Introduction

As protagonist members at the base of the food chain, marine eukaryotic phytoplankton (micro-algae), together with cyanobacteria, contribute significantly to the ecology of the marine ecosystem, in addition to contributing almost half of the oxygen in our atmosphere [1] and to half of global carbon fixation [2]. Whilst we may refer to eukaryotic phytoplankton as independent entities (i.e., as micro-algal cells), their functioning and very existence is largely, and in some cases, almost entirely dependent on their “intimate” interaction with bacteria. This close algal–bacterial interaction mainly occurs on the algal cell surface (i.e., phycosphere), which is thought to be important to their ecological success [3,4,5]. This is proven by the fact that very few eukaryotic phytoplankton species can be maintained in the laboratory for long periods without their bacterial symbionts (i.e., in axenic state). For example, Amin et al. [6] reported a mutual sharing of iron and fixed carbon between several species of eukaryotic phytoplankton and their bacterial symbionts, mainly with members of the genus *Marinobacter*. Other bacterial symbionts have been reported to utilize eukaryotic phytoplankton exudates as carbon and energy sources [7,8], whereas the phytoplankton could benefit through bacterial-mediated trace metal/nutrient bioavailability [9]. A study by Kazamia and colleagues [10] reported the supply of bacterial-produced vitamin B12 to the eukaryote host in exchange for fixed carbon.

In recent years, members of three major eukaryotic phytoplankton lineages (dinoflagellates, diatoms, and coccolithophores) have been found to harbour obligate and generalist hydrocarbon-degrading (hydrocarbonoclastic) bacteria, including novel taxa of these organisms [11,12,13,14,15]. Whilst the underlying basis and mechanism(s) of this association remains unresolved, there is evidence suggesting that the enrichment of hydrocarbons on phytoplankton (micro-algal and cyanobacterial) cell surfaces [16,17,18] or their biogenic synthesis by the phytoplankton [16,17,19,20,21,22] plays a crucial role. Regardless of biogenic synthesis or adsorption of hydrocarbons from the surrounding seawater environment, the phycosphere of eukaryotic phytoplankton cells could be regarded as an important micro-biotope in the marine water column, and possibly also in shallow sediments, to which hydrocarbon-degrading bacteria are attracted to.

Quite often, studies aimed at exploring the co-existence and relationship of bacterial symbionts with their eukaryotic phytoplankton hosts use methods that tease them apart and/or study them as independent entities. Taking into account the intimate relationship that exists between these organisms, and the fact that the degradation of hydrocarbons can be enhanced when bacteria and phytoplankton coexist e.g., [23,24], studies assessing phytoplankton-bacterial communities would be more relevant when these organisms are investigated together as a microbiological unit, as they exist naturally. Many of the hydrocarbonoclastic bacteria reported in the literature, from isolation techniques or through sequencing surveys, were quite possibly directly associated with eukaryotic phytoplankton cells in the original water samples at the time of their collection. The development of methods for rapid species-specific quantification of these bacteria with eukaryotic phytoplankton, and that can be applied directly to field samples, would greatly expand this field and enhance our understanding of these algal–bacterial associations in the ocean.

Flow cytometry is a quantitative sub-microscopic particle analysis method commonly used for counting cells and detecting other characteristics of individual particles [25]. The power of this method is enhanced when combined with fluorescence in situ hybridization (FISH-Flow), allowing for the identification and quantification of a range of cell types of different sizes and fluorescent characteristics. Accurate quantification of targeted bacterial taxa within communities has previously been achieved by this method [26,27,28,29]. More recently, FISH-Flow has been combined with cell sorting [30] and protein detection [31] for high-throughput multi-parametric measurement of various cell types. Populations of marine picoeukaryotes have also been quantified using FISH-Flow [32]. The application of combining Catalyzed Reporter Deposition (CARD) FISH with flow cytometry has also been explored [33] and later further improved [34] with a focus on the identification and quantification of specific types of bacteria. However, so far no reports have explored combining FISH with flow cytometry to examine bacterial communities, including hydrocarbonoclastic bacteria, living associated on the phycosphere of eukaryotic phytoplankton, and neither directly to assess this in natural seawater samples.

**Figure 1:**
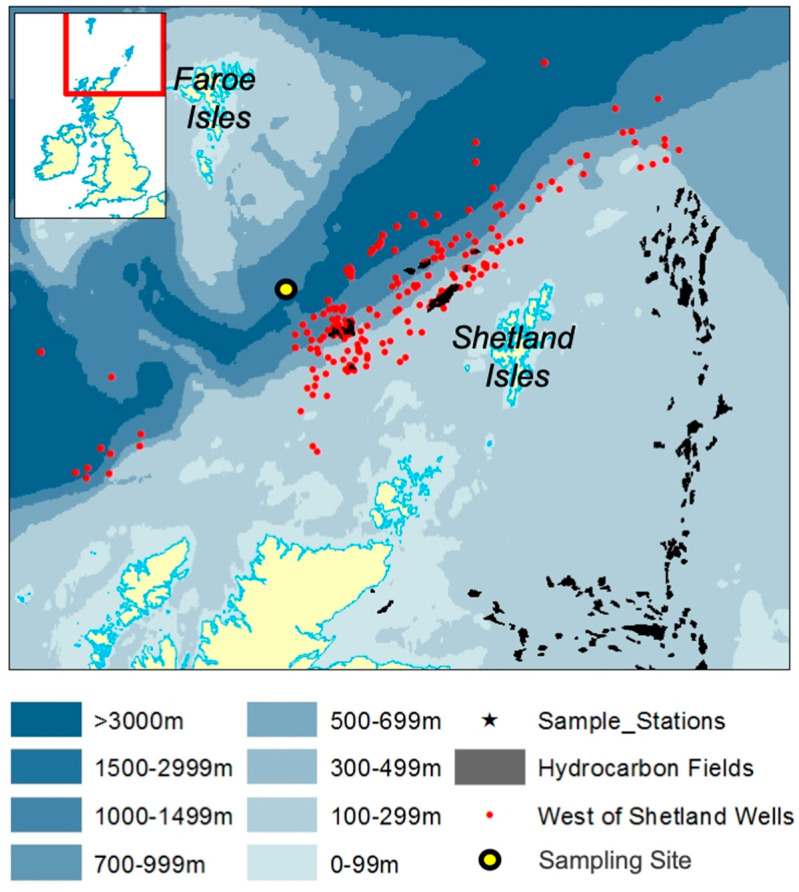
Map representing the Faroe-Shetland Channel showing bathymetry contours, hydrocarbon gas/oil wells and production fields, and the sampling location.

In the present study, we used FISH-Flow to detect and quantify members of the genus *Marinobacter* in sea surface waters of the Faroe-Shetland Channel—a deep and highly hydrodynamic region of the subarctic northeast Atlantic (Figure 1). The genus *Marinobacter* comprises members with hydrocarbon-degrading activities [35,36] and which are commonly found associated with species of marine eukaryotic phytoplankton constituting the three major lineages—diatoms, dinoflagellates, coccolithophores [6]. Recent work by our group has shown *Marinobacter* to be present at relative abundances of around 0.5% of the total bacterial community in surface waters of the Faroe-Shetland Channel, and which become enriched on marine oil snow (MOS) particles when it forms in the presence of crude oil [37]. We hypothesized that a subset of the total *Marinobacter* community in sea surface waters exists closely associated with particulate organic matter, including the eukaryotic phytoplankton population. To identify the majority of *Marinobacter* spp. in surface waters of the Faroe-Shetland Channel, we used a FISH probe set that targets up to 75% of this genus [38]. Applying this probe set with FISH-Flow, we report on the abundance and tight coupling of *Marinobacter* cells associated with eukaryotic phytoplankton communities in the Faroe-Shetland Channel and which we suspect may reflect a similar association of these organisms in surface waters elsewhere in the global ocean.

## 2. Materials and Methods

### 2.1. Study Site and Field Sampling

During a research cruise aboard MRV *Scotia* in April–May 2015, a sea surface phytoplankton sample was collected from a deep subarctic region called the Faroe-Shetland Channel (60°37.84′ N, 4°54.60′ W; water depth 1022 m). The Faroe-Shetland Channel is a dynamically complex region [39] that is the “spaghetti junction” of Icelandic, Norwegian and Atlantic currents, located approx. 200 miles north of Scotland’s most northerly point. The sampling site is located along the Fair Isle-Munken (FIM) line, which is a sampling transect that runs between the Faroe and Shetland Isles, north of Scotland. This site falls within a sea surface water mass defined as Modified North Atlantic Water (MNAW) which originates from the Faroe Islands and travels in a south-westerly direction through the Faroe-Shetland Channel before diverging south (Figure 1). MNAW is a warm and saltier Atlantic water mass compared to the underlying Arctic/Icelandic cold-water masses that are found at depths from 400 to 1500 m in the Faroe-Shetland Channel [39].

Sampling was performed using a phytoplankton net (50 μm mesh size) that was trawled 10 m below the sea surface (temp. 7.6 °C) for 20 min. Twelve equal volumes (1.5 mL) of the collected phytoplankton fraction were each gently centrifuged (300× *g*; 1 min.) to pellet the eukaryotic phytoplankton population. The supernatant fractions were collected and set aside at 4 °C. Each of the twelve phytoplankton pellets were washed once with filter-sterilized (0.2 μm) 0.05 M phosphate-buffered saline (PBS; pH 7.3) using the same gentle centrifugation step as before; the supernatant fractions from this wash step were combined with the first collected supernatant fractions stored at 4 °C. The resultant washed phytoplankton fractions were fixed with a filter-sterilized (0.2 μm) solution of 4% paraformaldehyde for 30 min at 4 °C. The fixed phytoplankton fractions were then each washed three times by centrifugation (8000× *g*; 10 min) with PBS to remove the fixative. The washed fractions were stored frozen onboard the ship at −20 °C in 1:1 (*v*/*v*) PBS and ethanol (1 mL total volume) for subsequent analysis by FISH-Flow upon return to the laboratory (described below).

Non-fixed samples of the trawled phytoplankton population, herein referred to as “Phyto”, were prepared in the same way as above, though without fixative, and stored frozen at −20 °C onboard. For the collected supernatant fractions above, these were combined (50 mL total volume) and filtered through a 0.2 μm Nuclepore filter (Whatman, Maidstone, UK) to collect the bacterial biomass representing the bacterial community loosely associated with the phytoplankton population, herein referred to as “Phyto_super”. All filters were stored at −20 °C onboard. Upon return to the laboratory, total genomic DNA was extracted from the frozen “Phyto” and “Phyto_super” filters for analysis by barcoded amplicon MiSeq sequencing (described below)—these samples, respectively, represented the bacterial community found tightly associated with the eukaryotic phytoplankton population, and the bacterial community that was loosely associated with the phytoplankton cells.

### 2.2. Barcoded-Amplicon Illumina MiSeq Sequencing and Bioinformatic Analysis

For DNA extractions, frozen filters were crushed into liquid nitrogen to a fine powder and DNA extracted according to the method of Tillet and Nielan [40] which utilizes chemical cell lysis with potassium xanthogenate buffer. Unfortunately, some of the replicates were lost due to a haphazard occurrence in the laboratory, but we were able to salvage and process two replicates of the “Phyto” and one of the “Phyto-super” samples. DNA extracts were resuspended in 20 μL of 1 mM TE buffer and stored at −20 °C for Illumina barcoded-amplicon sequencing. Extracted DNA was confirmed using a Nanodrop 3300 fluorescence spectrometer (Thermo, Waltham, MA, USA and further confirmed by gel electrophoresis. PCR amplifications of the V3-V4 16S rRNA gene fragment were performed on all genomic DNA using MyTaq polymerase (BioLine, Heidelberg, Germany), as described by the manufacturer’s protocol. Primer sequences of choice were the universal bacterial primers of forward or reverse direction 341F (CCTACGGGNGGCWGCAG) and 785R (GGACTACHVGGGTATCTAATCC) [41]. Target-specific primer pair coverage distribution was tested with TestPrime 1.0 tool against SILVA RefNR database [42]. Negative controls (molecular grade water) were employed to check for DNA contamination during the PCR procedure. PCR products were quantified by nanodrop (as above) and checked by gel electrophoresis. PCR products (of ~440 bp size) were enzymatically cleaned of polymerase and residual primers using 10 U *Exonuclease I* and 1 U FastAP (ThermoFisher, Waltham, MA, USA) according to the manufacturer’s protocol (ThermoFisher, Waltham, MA, USA).

Illumina MiSeq sequencing was performed at the University of Liverpool, Centre for Genomic Research. At the sequencing facility, the PCR products underwent a second round of PCR (8 cycles), adding barcodes and the Illumina Nextera XT adapters of the amplified sequences as per the adapted protocol of Berry et al. [43]. This two-round PCR approach has been shown to reduce PCR bias and sequencing artifacts, especially common for complex environmental multi-bacterial samples [43]. Prepared sequencing libraries were qualified and quantified using Qubit Fluorometer and Bioanalyzer systems (ThermoFisher, Waltham, MA, USA) and run on a 2 × 250 paired-end run of the Illumina MiSeq platform. De-multiplexed and primer trimmed data files were returned for downstream processing, as described below.

All reads were processed using Mothur and following the MiSeq SOP [44]. In brief, all sequences were trimmed to 420 bases or less and low-quality sequences were discarded. All reads were aligned to the 16S rRNA gene of *E. coli* (J01859.1) and trimmed to equal lengths. Each sequence was clustered to 97% similarity to form OTUs and a representative sequence from each OTU was compared to the Silva nr database (v138) to provide taxonomic identification. These data were standardized for read count, resulting in 16S rRNA relative abundance counts for each treatment. All nucleotide sequence data have been deposited in NCBI Sequence Read Archive (SRA) and are available under BioSample accession numbers SAMN16737052, SAMN16737053 and SAMN16737054.

### 2.3. FISH-Flow Procedures

#### 2.3.1. Preparation of Pure Cell Culture Controls for FISH-Flow

A pure culture of *Marinobacter algicola* (DSM 16394) was used as a positive control to test the FISH protocol and to calibrate the flow cytometer (BD LSR Fortessa, USA; see below) for the detection of *Marinobacter* in the collected field samples. For this, *M. algicola* was grown on marine broth medium (ZM/10) composed of ¾-strength naturally-aged seawater, peptone (0.05%), yeast extract (0.01%), and supplemented after autoclaving with filter-sterilized (0.2 μm) trace elements and vitamins to final concentrations as previously described [45]. Samples of the cells in the exponential phase were fixed with a filter-sterilized (0.2 μm) solution of 3% paraformaldehyde for 30 min at 4 °C. The fixed cells were then washed three times (8000× *g*; 10 min) using filter-sterilized (0.2 μm) 0.05 M PBS. The washed fractions were stored at −20 °C in 1:1 (*v*/*v*) PBS and ethanol (1 mL total volume) for FISH and subsequent calibration of the flow cytometer for the detection and quantification of *Marinobacter* (see below).

#### 2.3.2. FISH Protocol

Fixed samples of *M. algicola* cell suspensions and the twelve fixed phytoplankton field samples were thawed and washed three times with 0.05M PBS (8000× *g*; 10 min) at 21 °C. For hybridization, this was performed in solution using a modified version of the FISH-Flow protocol described by Nettmann et al. [28]. The samples were gently re-suspended in 221 μL of 46 °C preheated hybridization buffer, which contained 900 mM NaCl, 20 mM Tris/HCl (pH 7.2), 0.1% SDS, 25% formamide [38] and 21 μL of the *Marinobacter*-specific probes (50 ng μL^−1^ final concentration) Mrb-0625-a and competitor Hal-0625-a [38], or the non-sense probe NON338 [29]. Probe Mrb-0625-a (labeled at the 5′-end with the sulfoindocyanine dye CY3), the labeled NON338 and unlabeled Hal-0625-a probes were all obtained from IDT (Integrated DNA Technologies, Coralville, IA, USA). The *Marinobacter*-specific probe (Mrb-0625-a) currently targets > 62% of the *Marinobacter* genus and is used together with the competitor probe to block hybridization to six *Halomonas* species which share a one base pair mismatch [38]. Hybridisations were also performed in the absence of these probes in order to subtract any autofluorescence as background during the subsequent analysis of these samples by FISH-Flow. Samples were incubated at 46 °C for 2 h whilst inverting periodically. Immediately following hybridization, samples were centrifuged (as before), the supernatant fractions discarded, and then 500 μL of preheated (48 °C) washing buffer (0.149 M NaCl, 20 mM Tris/HCl (pH 7.2), 5 mM EDTA) was added to the samples. This washing buffer maintained slightly more stringent conditions than hybridization buffer. Samples were incubated for 15 min at 48 °C prior to washing twice with 0.05 M PBS (pH 7) [28] and maintained at 4 °C for immediate microscopic observation and analysis by FISH-Flow. To confirm successful hybridization, hybridized samples of *M. algicola* (as positive control) were visualized using a Zeiss epifluorescence microscope (Axio Scope.A1) equipped with a Zeiss digital fluorescence imaging camera (AxioCam MRm).

#### 2.3.3. Flow Cytometry

Hybridized samples for flow cytometry were counterstained with 4′,6-diamidino-2-phenylindole (DAPI) to a final concentration of 1 μg mL^−1^, following standard methods in order to identify DNA containing species [46]. The samples were then passed through a 70 μm nylon mesh filter to remove cell aggregates that may cause blockage of the flow cytometer during analysis. For determining the relative size of particles in the samples, the flow cytometer was set to the most useful scatter profile (Forward Scatter FSC vs Side Scatter FSC) by using size-standardized beads covering a range of 1.3 μm to 15 μm (Thermo Fisher Scientific, Waltham, MA, USA).

Samples were analysed using a BD LSR Fortessa Flow Cytometer with five lasers (λ_ex_: 355 nm, 405 nm, 488 nm, 561 nm and 640 nm) and 18 fluorescence detection units, in addition to the parameters Forward Scatter (FSC) and Side Scatter (SSC). FSC is defined as the scattered light in the forward direction, with the signal proportional to cell size. SSC is defined as the signal intensity (measured at 90° to the FSC signal) and is proportional to cell complexity/granularity. Scatter profiles (FSC and SSC) were optimized by measuring the background using filtered solutions (0.22 μm for Sheath fluid; 0.1 μm for dH_2_O and PBS) and size calibration beads in order to accurately gauge the size and complexity of each cell in the samples. The phycoerythrin (PE) channel was used for Cy3 fluorescence (λ_ex_ = 561 nm (yellow/green laser) and a 585/15 nm emission band-pass filter), whilst the peridinin-chlorophyll protein (PerCP) channel was used to measure chlorophyll (Chl) as auto-fluorescence emitted by eukaryotic phytoplankton (micro-algal) cells (λ_ex_ = 488 nm [blue laser] and a 670/14 nm emission band pass filter). Spectral overlay of the Cy3 into the Chl channel, and vice versa, was compensated by using a sample of Cy3-labelled *M. algicola* and non-stained marine eukaryotic phytoplankton sample for chlorophyll auto-fluorescence. DAPI was excited by a 355 nm UV laser and measured using an emission filter 450/50 nm.

#### 2.3.4. Data Processing and Statistical Analysis

For each particle/cell that passed through the flow cytometer, information from all five signals (FSC, SSC, Cy3, Chl and DAPI) of the above channels/lasers was collected and saved as a FCS (Flow Cytometry Standard) file. Using De Novo Software FlowJo V10 (FlowJo, LLC, USA), the following gating strategy was implemented to quantify the target population of total eukaryotic phytoplankton cells, and subsequently those with associated signals for *Marinobacter* cells (Cy3). Using plot 1 (see below) created above, fluorescence intensity of chlorophyll-*a* (PerCP Channel, 670 nm emission) was plotted against FSC. Eukaryotic phytoplankton cell signals (Big Chl+) were discriminated using chlorophyll fluorescence (Chl) and “big” size (FSC), as determined using *M. algicola* alone as Chl-negative control (treatment E; Table S1). The gated population “Big Chl+” was further analysed to identify and quantify Cy3 fluorescence signals when plotted against DAPI signals. Analysing the negative control treatments C and D together with the positive control treatment B for *M. algicola* cells (Table S1), positive Cy3 signals were observed. Particles of interest were labelled Cy3+ DAPI+ and gated, representing the presence of *Marinobacter* together with eukaryotic phytoplankton. Using this gating strategy, the frequency (%) of *Marinobacter* that were within the Big Chl+ gate was determined.

One-way ANOVA was used with Tukey’s post hoc test to compare groups using Statistical Package for the Social Sciences (SPSS). Student’s *t*-tests were used for comparing between two groups (Treatments A, B, C and D; Table S1) using SPSS. When necessary, data were log_10_-transformed in order to conform to parametric assumptions of normality and homogeneity of variance.

## 3. Results and Discussion

### 3.1. MiSeq 16S rRNA Sequencing Analysis of the Bacterial Community Associated with the Eukaryotic Phytoplankton Population in the FSC

**Figure 2:**
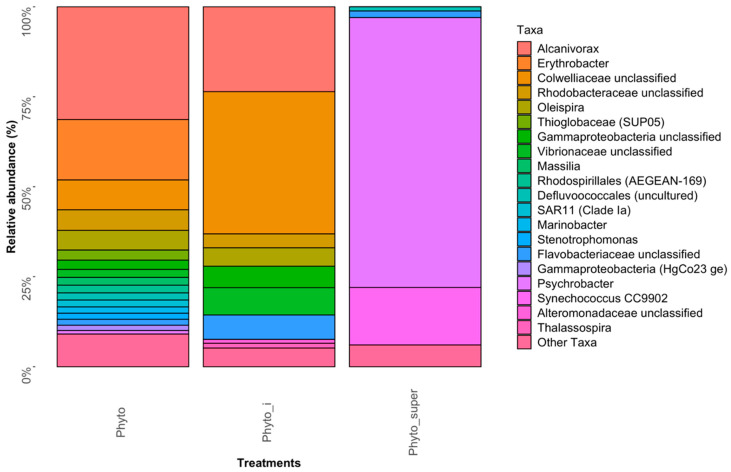
Abundant bacterial phyla in the “Phyto” and “Phyto-super” fractions.

The “Phyto” bacterial community structure—i.e., that found tightly associated with the eukaryotic phytoplankton cell population in surface waters of the FSC—was dominated by *Alcanivorax* and members of unclassified *Colwelliaceae*—40–63% combined contribution to the total 16S rRNA gene sequence reads in both “Phyto” libraries (Figure 2; Table S1). Minor contributions, between 1–5% of relative abundance, were observed for *Thioglobaceae*, *SAR11*, *Oleispira*, *Vibrionaceae*, *Psychrobacter*, *Marinobacter*, *Massilia*, *Erythrobacter*, *Stenotrophomonas*, *Thalassospira*, and members of the order *Rhodospirillales* (Marine Group AEGEAN-169) and unclassified *Flavobacteriaceae*, *Rhodobacteraceae*, *Gammaproteobacteria*, *Pirellulaceae* and *Alteromonadaceae*. This bacterial community profile reflects that typically found associated with eukaryotic phytoplankton from laboratory cultures and in the field [5,47,48,49], including both obligate and putative oil-degrading taxa, and substantiates our methodological approach for isolating the phytoplankton population collected from the field by net trawl. *Alcanivorax* are often strongly selected for in oil-impacted environments [35,36] and have been found associated with marine phytoplankton [47]. The family *Colwelliaceae* is represented by four genera, which includes *Colwellia*—a genus of psychrophilic marine heterotrophic generalists [50]. However, non-psychrophilic members of *Colwellia*, able to degrade PAHs, were identified in sea surface oil slicks in the Gulf of Mexico during the active phase of the Deepwater Horizon oil spill [51]. During this spill, *Colwellia* had rapidly become enriched in sea surface oil slicks and the subsurface oil plume [51]; some members of the genus were shown to having a predilection for degrading and growing on the dispersant Corexit and crude oil [52]. In surface waters of the FSC, we previously reported strains of the genus as susceptible to hydrocarbons and/or to a synthetic chemical dispersant used to treat oil spills [37]. In a more recent report, we showed *Colwellia* from the FSC as a member of the bacterial community associated with marine oil snow (MOS) [53], similarly to a hydrocarbon-degrading *Colwellia* strain, RC25, that was isolated from deep waters in the Gulf of Mexico following the Deepwater Horizon oil spill and shown to dominate the bacterial community associated with MOS [54]. Our results and those of past studies suggest that some members of the genus have an affinity for planktonic organic matter, such as MOS (i.e., marine snow containing oil) or with eukaryotic phytoplankton cells (this study).

A number of organisms associated with the eukaryotic phytoplankton population of the FSC were also identified in a previous study to be associated with a phytoplankton population in surface waters of the west coast of Scotland and which had become enriched by crude oil [55]. These organisms included members of *Rhodobacteraceae*, *Thalassospira*, *Cycloclasticus*, *Oleispira*, *Flavobacteriaceae*, and *Verrucomicrobium*. Of these taxa, *Thalassospira* contain members with reported hydrocarbon-degrading activity [56], and *Oleispira* is a genus comprising members of obligate hydrocarbon-degraders with a preference for utilizing straight-chain aliphatics [35,36]. The family *Rodobacteraceae* includes the *Roseobacter* clade, which is commonly found in high abundance during algal blooms [57] and is recognized for encoding multiple ring-cleaving pathways that participate in the degradation of monocyclic and PAHs [58]. The *Flavobacteriaceae* contain members with hydrocarbon-degrading activity, such as *Arenibacter* (not detected in this study) which is a genus comprising members with the ability to degrade PAHs [15]. Our results show that the autochthonous population of eukaryotic phytoplankton, of cell size 50–70 μm, in surface waters of the sampled region in the northeast Atlantic, is a natural source for a rich diversity of oil-degrading bacteria. It could be assumed that this population of phytoplankton may act as an important seed source of oil-degrading bacteria, of which some would be expected to respond strongly in the event of an oil spill in this region where there exists a high presence of oil and gas extraction activity.

Of the less represented phyla found tightly associated with the phytoplankton population of the FSC, a few are worth mentioning. *Marinobacter* was found almost entirely associated with the “Phyto” sampled fraction (up to 1.75%), as its presence in the “Phyto_super” library was insignificant with only two *Marinobacter* sequences. As discussed below, this 1.75% relative cell abundance of *Marinobacter* found tightly bound to the phytoplankton population corroborates the results we subsequently found using FISH-Flow. Though its relative abundance in the “Phyto” libraries was <1%, the detection of members of the family *Porticoccaceae* is also notable as only two genera are included in this family, one of which is represented by *Porticcoccus hydrocarbonoclasticus*—an obligate hydrocarbon degrader recognized for having an almost exclusive preference for polycyclic aromatic hydrocarbons (PAHs) as the sole source of carbon and energy [12,59]. The detection of *Methylophaga* sequences in the phytoplankton community, albeit <0.1% relative abundance, is also notable as these organisms were also found associated with a natural phytoplankton community in a previous study where they were shown to become significantly enriched by crude oil [55]. Members of this genus are recognized for their almost exclusive requirement for C_1_ sources (e.g., methanol, methylamine, dimethylsulfide) as sole growth substrates, with the exception of some strains that are also capable of metabolizing fructose [60]. However, employing DNA-based stable-isotope probing, Mishamandani et al. [61] provided substantiated evidence implicating members of this genus in also being able to utilize hydrocarbons as a sole source of carbon and energy.

The “Phyto_super” community structure, representing the bacterial population loosely associated with the eukaryotic phytoplankton cell population in surface waters of the FSC, was largely dominated by *Psychrobacter* and, to a lesser extent, *Synechococcus*—respectively contributing 75% and 15.9% to the total 16S rRNA gene sequence reads in both “Phyto_super” libraries (Figure 2; Table S1). Minor contributors were identified to belong to *Pirellulaceae* of the Planctomycetes and unclassified *Flavobacteriaceae* (collectively 3.8%). Members of all these phyla have been found associated with laboratory cultures of eukaryotic phytoplankton or with phytoplankton blooms in the field [5,47,49]. Therefore, they can be assumed to have been loosely associated with the phytoplankton cell population collected by the net trawl and had become dissociated during its manipulation.

Our identification of *Synechococcus* associated with the eukaryotic phytoplankton population (15% relative abundance of total bacterial community) is the first report documenting the association of these organisms in a region of the northeast Atlantic. The significance of this follows a recent report by Lea-Smith et al. [17], where the authors discovered the production of alkanes, predominantly pentadecane and heptadecane, by *Synechococcus* and *Prochlorococcus* [17]—the two most abundant cyanobacteria in the global ocean [62]. Like these cyanobacteria, some micro-algae from the sunlit, near-surface ocean are also known to produce hydrocarbons, suggesting that these organisms collectively produce a highly significant hydrocarbon input to the global ocean. This is believed to support communities of hydrocarbon-degrading bacteria, even in the absence of any obvious hydrocarbon contamination [63]. In the study by Lea-Smith et al. [17], the cyanobacterial hydrocarbons alone were calculated to provide an input of 308–771 million tons (280–699 million metric tonnes) of pentadecane and heptadecane to the sea per year—a quantity of hydrocarbons that is ~1% of bioavailable dissolved organic carbon in the ocean. Indeed, the abundance and ubiquity of cyanobacteria in the marine environment suggest hydrocarbon production in the oceans could be considerable and broadly distributed geographically. In the laboratory, the authors showed that these hydrocarbons supported the rapid growth of the *n*-alkane-degrading species *Alcanivorax borkumensis*.

As mentioned, members of *Alcanivorax* have been reported associated with laboratory cultures of marine micro-algae [47], and here in the present study we show for the first time that they are tightly associated with a natural community of eukaryotic phytoplankton in the field. Notably, their abundance was much higher (up to 31%) than expected and reported in seawater environments where there is no obvious petrochemical contamination. However, the FSC is a body of water that for many years has been an oil-producing region. Hence, small discharges from industry activity, as well as frequent shipping and oil transportation activities in the region, could be a continued source of petroleum into this region that feeds a higher-than-normal population of hydrocarbon-degrading bacteria. Though no confirmed oil seeps are known along the seabed of the FSC, nor in adjacent water bodies such as the North Sea, satellite surveys suggest the presence of subsurface oil seeps on the east and west of Scotland and offshore in the North Sea (Peter Browning-Stamp, pers. comm.). Such persistent surface oil slicks suggest naturally occurring oil seepage, which may contribute to elevated (above background) levels of an oil-degrading bacterioplankton population, including *Alcanivorax*, in the FSC; a similar situation is well documented in the Gulf of Mexico [64]. However, we do not exclude the likelihood that such a community is in part also supported by the close coupling of these organisms with hydrocarbon-producing phytoplankton, such as *Synechococcus*.

### 3.2. FISH-Flow Optimisation and Methodological Considerations

In the present study, FISH-Flow was employed with probe MRB625a to identify *Marinobacter* cells associated with the eukaryotic phytoplankton population (of cell size >50 μm) in sea surface waters of a region in the northeast Atlantic. With FISH, there is an inherent possibility of nonspecific binding. However, the significant differences between negative controls and positive controls demonstrate the hybridization worked effectively and that nonspecific binding levels were within acceptable standards. This provided confidence that the Cy3 channel fluorescence was attributed to the probe (MRB625a), and that detected *Marinobacter* signals were very closely associated with larger eukaryotic phytoplankton population.

Whilst we present here quantitative data for *Marinobacter* cells found tightly or loosely associated with a natural community of eukaryotic phytoplankton, we are aware that this is not an accurate representation of the complete phytoplankton community in the sea surface sample collected at the time from the northeast Atlantic. However, this was unavoidable as it was necessary to use a 70 μm size nylon mesh filtration step to remove large (>70 μm) particulates from the 50 μm net trawl sample in order to avoid clogging of the flow cytometer’s flow cell—hence, the seawater sample that was used for FISH-Flow contained cells (and other particulates) of size range 50–70 μm. Whilst our analysis also did not account for the possibility of finding *Marinobacter* associated with the eukaryotic phytoplankton population of cell sizes <50 and >70 μm, as the pore size of our net trawl was 50 μm and we used a >70 μm pre-FISH-Flow filtration step, our aim was principally to determine whether these bacteria are found associated with natural communities of eukaryotic phytoplankton in sea surface waters.

Some authors have in the past reported issues with clogging of their flow cell due to the presence of large aggregates, such as when using flow cytometry coupled with CARD-FISH to quantify nanoplankton and picoplankton communities [32,33,65,66]. Such studies also reported cell loss due to multiple centrifugation steps and re-suspension during hybridization. Our methodology attempted to minimize these shortcomings by reducing the number of centrifugation steps and the processing time using a modified version of the Nettmann et al. [28] protocol (without catalyzed reporter deposition). Gentle centrifugation was intended to maintain the natural microbial assemblage as much as possible in order to obtain a near accurate representation of the *Marinobacter* population associated with the eukaryotic phytoplankton community, albeit represented by the population of size range 50–70 μm.

### 3.3. FISH-Flow Identification of Marinobacter Associated with the Eukaryotic Phytoplankton Population

**Figure 3:**
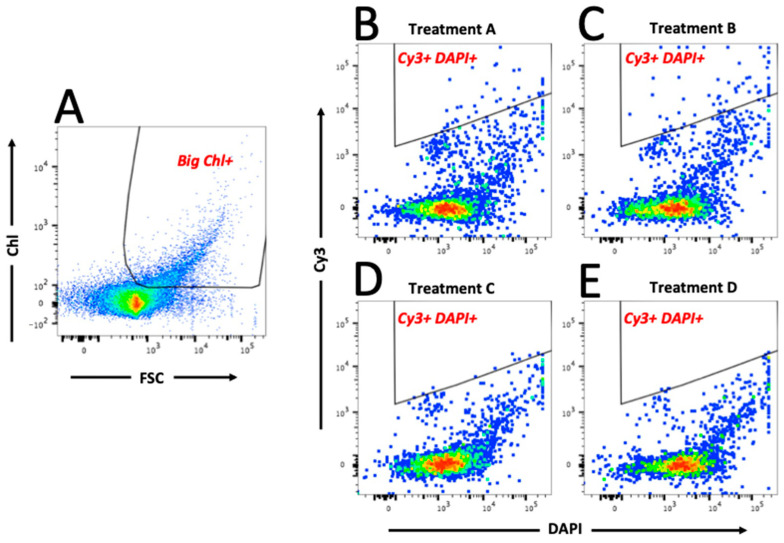
Bivariate plot and gating sequence using scatter (FSC) and chlorophyll (Chl) parameters to gate big eukaryotic phytoplankton ((**A**) Big Chl+), followed by fluorescence (DAPI and Cy3) parameters to gate viable (DAPI+) *Marinobacter* cells (Cy3+) in gate (**B**–**E**) Cy3+ DAPI+ (examples from treatments A–D). Gate names are shown in red text on each plot. Treatment A represents the field sample data, whereas treatments B represents the positive (*M. algicola* enrichment) control. Non-sense probe and negative (probe absent) control treatments (C and D, respectively) clearly contain less Cy3 fluorescence in gate “Big Chl+”. The red, green and blue colouration in each plot represents the signal intensity, from strong to weak respectively.

**Figure 4:**
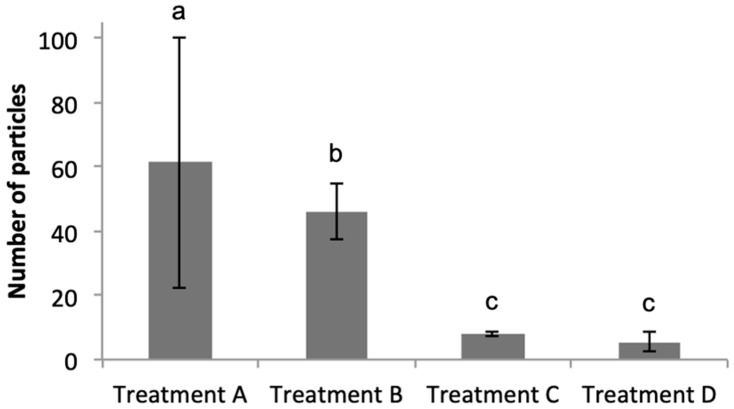
Total number of particles (mean values and standard deviation for three replicates) in gate “Cy3+ DAPI+” of Figure 2 comparing all treatments A–D. Lower case letters show the results of Tukey’s post hoc test: a treatment sharing the same letter with another treatment denotes the treatments were not significantly different from each another. See Figure 3 caption for key to treatments A–D.

As shown in Figure 3A, the eukaryotic phytoplankton population (“Big Chl+”, left hand plot) were first gated, and subsequently data from this gate were analysed to identify the *Marinobacter* population by plotting Cy3 (y-axis) versus DAPI (x-axis)—shown in the “Cy3+ DAPI+” gated regions of Figure 3B–E; total particles in “Cy3+ DAPI+” gates are also shown. As expected, the negative control treatments (C and D) showed a clear lack of particles in these gates, and log_10_-transformed data confirm that the number of particles were significantly lower (*p* < 0.01) compared to that in treatments A and B. Results of a Tukey’s post hoc test (Figure 4) show that in Treatment A, which represents the field sample data, there were 60.4 ± 38.7 particles in the Cy3+ DAPI+ gate (Figure 3B); significantly more compared to the 7.59 ± 0.49 particles in the non-sense treatment C. In Treatment A, a total of 1.53 ± 1.33% eukaryotic phytoplankton cells (Big Chl+) tested positive for the *Marinobacter* Cy3 signal. These results confirm the close physical attachment of *Marinobacter* on the phycosphere of about 1.5% of the eukaryotic phytoplankton community (of cell size >50 μm) in sea surface waters of the northeast Atlantic during the time of sampling in the spring of 2015. This *Marinobacter*-phytoplankton association is consistent with studies that have explored this with laboratory cultures of marine micro-algae [6,67], and supports the view of *Marinobacter* as ubiquitous microorganisms in the marine environment [35,36].

Whilst *Marinobacter* were found tightly associated with ~1.5% of the eukaryotic phytoplankton community in surface waters of the Faroe-Shetland Channel that may be assuming symbiotic relationships between these organisms [6], other *Marinobacter* cells were found associated with 2.19 ± 0.64% of the total particulate fraction (of <50 μm size) exhibiting no chlorophyll-*a* signals. This population may be involved in nutrient cycling, as has been reported for this genus [67,68]. We also identified a large population of *Marinobacter* cells (54.5 ± 1.6%) that were not associated with eukaryotic phytoplankton or other particulate matter. It should be noted that during flow cytometry, individual particles identified of size <1 μm can be assumed to be cells that were loosely attached to larger particles (i.e., eukaryotic phytoplankton cells or other non-chlorophyll-containing particulate matter) originating from the field samples and that had become detached considering the use of a 50 μm mesh net to collect the phytoplankton field sample. We assume that this population of *Marinobacter* cells that were not detected as epibiotic on eukaryotic phytoplankton cells may have originated from the phycosphere, and likely were loosely associated and consequently became detached during the FISH-Flow processing steps.

Interestingly, some of the highest Cy3 fluorescence intensities were found on large eukaryotic phytoplankton cells. Possibly individual phytoplankton cells contained high numbers of epibiotic *Marinobacter* cells attached to them. Alternatively, since with FISH the fluorescence intensity is an indicator of ribosomal abundance inside of cells, and therefore also metabolic activity [26,28], we can assume that this population of *Marinobacter* cells producing high Cy3 intensities were highly active at the time of our sampling—i.e., 1.55 ± 1.33% of the larger-sized (>50 μm) eukaryotic phytoplankton cells. This is supported by the fact that the mutual symbiosis of *Marinobacter* and eukaryotic phytoplankton is, at least in part, mediated by the sharing of iron and fixed carbon [6].

## 4. Conclusions

Members of the genus *Marinobacter* are known as ubiquitous, opportunistic heterotrophs [67] in marine environments that can use a wide range of substrates [69,70]. These organisms are most widely known for their ability to utilize hydrocarbons and in responding positively to marine oil spills [35,36,69]. Their distribution in the marine environment is largely confined to oligotrophic (i.e., nutrient-/substrate-limited) waters which make up at least 18% (estimated oligotrophic gyre area [71,72]) of the global ocean. Species of *Marinobacter* may thus be well adapted to survive long periods of low nutrient/substrate availability [67,73,74], especially for species that exist in a free-living state. However, using FISH-Flow and MiSeq sequencing, we have found that the total *Marinobacter* population in surface waters of the FSC is apparently tightly bound to the eukaryotic phytoplankton population of cell size between 50–70 μm. Isolated strains of this genus have been found living associated on the phycosphere of laboratory cultures of phytoplankton as part of a mutual symbiosis [6]. This adaptation of *Marinobacter* to physically coupling together with phytoplankton cells may play importantly to its advantage in oligotrophic waters compared to bacteria that exist in a free-living state where such organisms will be limited by the availability of nutrients. The phycosphere offers what can be described as a protective habitat and a source of nutrients provisioned by the algal cell partner, such as algal exudates (vitamin B12, fixed carbon) as carbon and energy sources [6,7,8,10]. This is supported by the fact that very few phytoplankton species can be maintained for long periods in an axenic state in the laboratory.

In conclusion, this work highlights FISH-Flow as an effective method for detecting and quantifying hydrocarbonoclastic bacteria on eukaryotic phytoplankton in seawater samples, and for potentially other epibiotic bacteria where a FISH probe set is available. Using multiple probes for differential targeting of bacterial groups would enable their rapid quantification in water samples, including their association with different size populations of eukaryotic phytoplankton and other planktonic particulate matter such as marine snow and marine oil snow (MOS) where Marinobacter (and other hydrocarbonoclastic taxa) have been found associated [37,75]. Whilst our sampling was limited to the spring 2015 and from one location in the global ocean targeting a specific size range (50–70 μm) of the eukaryotic phytoplankton population, our findings show for the first time the diversity and close association between hydrocarbon-degrading bacteria with a natural population of eukaryotic phytoplankton. Future work is planned to explore this in other ocean sites and across seasons of the year.

## Supplementary Materials

The following are available online at https://www.mdpi.com/2076-2607/8/12/1955/s1, Table S1: Probes used and their purpose for FISH.
