# Supplementary material for: Hydrocarbon-Degrading Bacteria Found Tightly Associated with the 50–70 μm Cell-Size Population of Eukaryotic Phytoplankton in Surface Waters of a Northeast Atlantic Region
Source: Microorganisms. 2020 Dec 9;8(12):1955. doi: 10.3390/microorganisms8121955 (PMC7763645; doi:10.3390/microorganisms8121955)
Supplement: Supplementary file 1 [file microorganisms-08-01955-s001.pdf]

**Supplementary table 1.** Probes used and their purpose for fluorescence *in situ* hybridisation (FISH) in the field sample treatments (A, B, C and D) and for the *Marinobacter algicola* control sample (treatment E).

| Treatment | Hybridization and probe selection | Replicates | Purpose |
|---|---|---|---|
| A | MRB625+Cy3; HAL625c | 3 | Experimental sample |
| B | MRB625+Cy3; HAL625c; enriched with fixed *M. algicola* | 3 | Positive control |
| C | NON338+Cy3 | 3 | Nonspecific binding quantification |
| D | probe absent | 3 | Autofluorescence quantification |
| E | *M. algicola* PFA-fixed culture MRB625+Cy3; HAL625c | 1 | Validation of MRB625 probe |
